# Supplementary figures and images for: Menstrual hygiene management interventions and their effects on schoolgirls’ menstrual hygiene experiences in low and middle countries: A systematic review
Source: PLoS One. 2024 Aug 22;19(8):e0302523. doi: 10.1371/journal.pone.0302523 (PMC11340951; doi:10.1371/journal.pone.0302523)

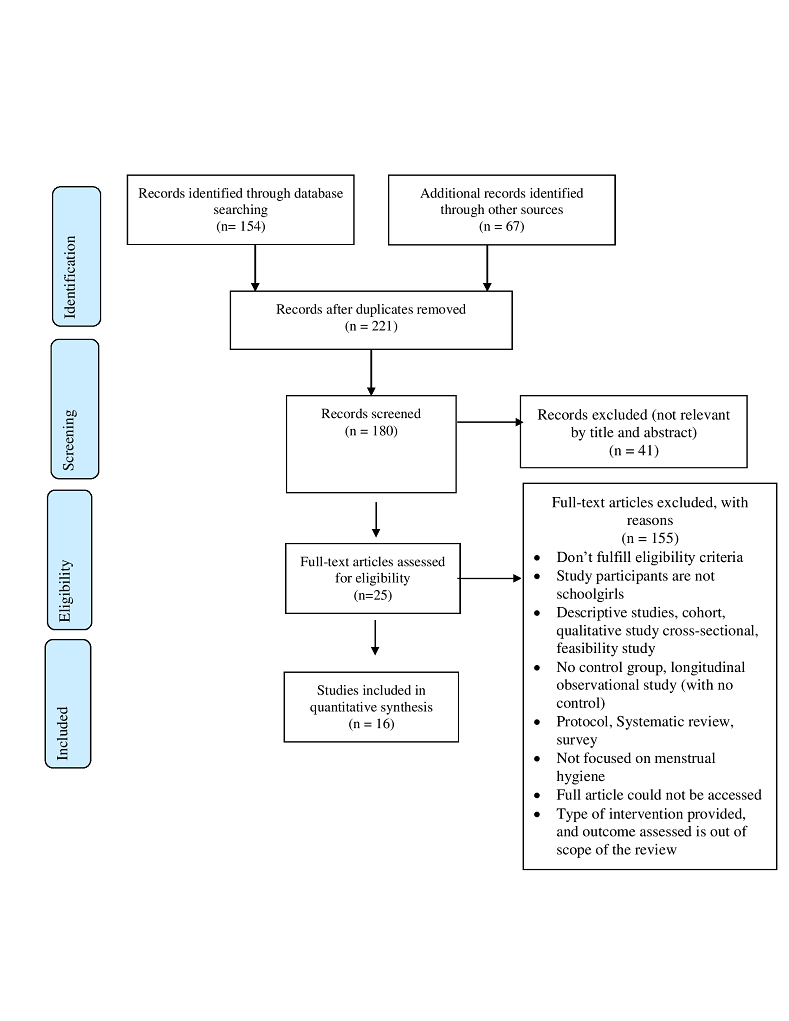

Supplement: S1 Fig — (TIFF) [file pone.0302523.s002.tiff]
