# Supplementary material for: Menstrual hygiene management interventions and their effects on schoolgirls’ menstrual hygiene experiences in low and middle countries: A systematic review
Source: PLoS One. 2024 Aug 22;19(8):e0302523. doi: 10.1371/journal.pone.0302523 (PMC11340951; doi:10.1371/journal.pone.0302523)
Supplement: S1 Protocol — (DOCX) [file pone.0302523.s003.docx]

**Menstrual hygiene management interventions and its effect on schoolgirls’ menstrual hygiene experience in low and middle countries: Protocol for systematic review**

Contents

[Introduction 3](#_Toc49593693)

[Objective 5](#_Toc49593694)

[Methods 5](#_Toc49593695)

[Study design 5](#_Toc49593696)

[Inclusion criteria 5](#_Toc49593697)

[Exclusion criteria 7](#_Toc49593698)

[Source Information and Search strategy 7](#_Toc49593699)

[Data management and selection process 8](#_Toc49593700)

[Data extraction process 8](#_Toc49593701)

[Risk of bias in individual studies 8](#_Toc49593702)

[Data Synthesis 9](#_Toc49593703)

[Discussion 9](#_Toc49593704)

# **Introduction**

Menstrual hygiene management (MHM) is defined as “women and adolescent girls using a clean menstrual management material to absorb or collect menstrual blood, that can be changed in privacy as often as necessary for the duration of a menstrual period, using soap and water for washing the body as required, and having access to safe and convenient facilities to dispose of used menstrual management materials” ([1](#_ENREF_1)). Sommer has also complimented issues of dignity and understanding stating that “They understand the basic facts linked to the menstrual cycle and how to manage it with dignity and without discomfort or fear” ([2](#_ENREF_2)). Accordingly, proper MHM requires clean absorbents; adequate frequency of absorbent change; washing the body with soap and water; adequate disposal; privacy for managing menstruation; access to information, and managing it in a dignified way.

Near to 52 % global female population is of reproductive age, while 500 million women and girls lack adequate facilities for menstrual hygiene management ([3-5](#_ENREF_3)). Moreover, multi-country assessment of MHM in Africa indicates that 70-72 % of girls reach menarche lacking knowledge and information as most of the adversities are related to lack of information and knowledge ([6](#_ENREF_6), [7](#_ENREF_7)). Lack of knowledge and guidance; poor access to menstrual hygiene supplies and materials; lack of privacy and access to water; unfavorable environment; socio-cultural and religious restrictions and taboos all together leaves girls to experience menarche and menstruation with fear, confusion and lack of confidence ([8-10](#_ENREF_8))

Shreds of evidence have shown that poor MHM predispose girls to school dropout, absenteeism, and low school performance ([11-14](#_ENREF_11)). In Sub-Saharan Africa, 20 % of school time is missed over a year due to menstruation-related issues ([4](#_ENREF_4)). However, menstruation is associated not only with education but it also with girls’ life in many aspects. These include; (1) physical health like reproductive and urinary tract infections and menstrual pain; (2)social restrictions including school absenteeism, school performance, and participation; (3) psychosocial impacts like shame, anxiety, insecurity and fear of stigma low confidence and dignity and quality of life in general ([15-19](#_ENREF_15)). These problems are attributed to multiple factors.

UNICEF has outlined the attributable factors in the theory of change for menstrual hygiene management challenges to include; 1) weak enabling environment, including a lack of political will, lack of policy framework, and lack of resource allocation. 2) Insufficient knowledge, guidance, and skills. 3) Inadequate access to basic WASH services and MHH supportive systems in schools, households, and health facilities. 4) Inadequate access to affordable and appropriate menstrual materials. 5) Unsupportive and patriarchal attitudes and social norms around menstruation leading to stigma, myths, and taboos ([20](#_ENREF_20)). Thus, it calls up for a coalition of multiple stockholders as boldly indicated by the MHM in ten agenda ([21](#_ENREF_21)).

'MHM in Ten agenda' is an agenda that has been advocated in 2014 with a goal that promotes; “Girls in 2024 around the world are knowledgeable about and comfortable with their menstruation and able to manage their menses in school in a comfortable, safe, and dignified way” ([21](#_ENREF_21)). This goal is believed to play a pivotal role in minimizing the gender gap in education by addressing the aforementioned problems experienced by girls and women. Hence, it would directly or indirectly contribute the realization of sustainable development goals (SDGs) including (a) good health and well-being (SDG 3); (b) inclusive and equitable quality education (SDG 4); (c) gender equality and women’s empowerment (SDG 5); (d) clean water and sanitation (SDG 6); and (e) economic growth, productive employment and decent work for all (SDG 8) ([22](#_ENREF_22)). Ethiopia has also developed a guide line for MHM in 2016 promoting multispectral engagement to address fife main issues. These include 1) human right issue; 2) safe MHM practice to prevent infection and odor; 3)reducing school absenteeism; 4) empower woman and increase their productivity; 5) and achievement of SDGs ([23](#_ENREF_23)).

To address the problems of MHM and realize SDGs, different modalities of interventions that are categorized as hardware and software interventions were postulated and experimented to appreciate its impacts on girls physical psychosocial wellbeing, MHM behavior (knowledge attitude and practice), school attendance and school performance([24-27](#_ENREF_24)). However, the impact is controversial especially on the intervention modalities and combination of hardware and software interventions.

There are controversial study findings about the effect of hardware interventions on menstrual KAP and school attendance. An experimental study done by Oster & Thornton portrayed that provision of menstrual cup to girls did not bring significant change in school attendance. Moreover, according to this study, menstruation-related school absenteeism is not statistically significant ([24](#_ENREF_24)). While Montgomery *et.al* in their experimental study showed that providing low-cost menstrual pad has a promising effect on school attendance of menstruating girls ([26](#_ENREF_26)).

Other similar experimental studies that investigated the impact of software intervention like puberty health education proved that it has heightened the MHM behavior (KAP) of girls ([25](#_ENREF_25), [27](#_ENREF_27)). Moreover, a combination of the two modalities of intervention is warranted to bring the desired outcome among girls ([26](#_ENREF_26)).

So far there is one systematic review done that is related to this review five years ago ([15](#_ENREF_15)). There has been increasing attention paid to MHM like the MHM in ten agenda. This global initiative calls for a cross-sectorial collaborative response that paves the way for many school-based interventions to close the gender gap in education. Hence, there are a number of studies since then. Moreover, to attain the goal of the MHM in ten agendas, there is a need for providing empirical evidence on MHM related school dropout, achievement, self-esteem, self-efficacy, sexual and reproductive health, and girls' inequity ([28](#_ENREF_28)). Therefore, this review will provide up-to-date evidence on how these multispectral initiatives have impacted girl’s MHM behavior, wellbeing, and schooling.

Moreover, the current review differs in context and population that it is focused on schoolgirls, excluding community-based interventions unlike the previous review ([15](#_ENREF_15)). This is to allow for more objective attributions of intervention to the setting and minimizing heterogeneity of interventions.

# **Objective**

The objective of this review is to synthesize the effect of school-based interventions on knowledge, attitude and practice of menstrual hygiene management, psychosocial, and physical wellbeing, school attendance, and performance among school girls, 2020

# Methods

## Study design

The proposed systematic review will be reported in accordance with the reporting guidance pro- vided in the Preferred Reporting Items for Systematic Reviews and Meta-analyses (PRISMA) statement ([29](#_ENREF_29)).

## Inclusion criteria

Studies to be included in this review will be selected based on the following criteria; participants, interventions and comparators, out-comes of interest, and type of study ( study designs) (PICOTS) ([30](#_ENREF_30)). Because of the difficulty of conducting both individual and clustered randomized control trials, there is a dearth of information, hence, other study designs like quasi-experimental or non-randomized trials, before and after studies will be included. The inclusion criteria for the participants in the review are adolescent or youth schools girls, university, or college female students. The intervention sought is MHM intervention (detailed in the intervention and setting section). The comparator will be girls who did not receive the indicated interventions. The outcomes of interest are MHM KAP, psychosocial, and physical wellbeing, school attendance, school dropout and school performance of the girls. The setting under which his review takes into consideration is school and community; where a school is conceptualized as either college, university, high school, middle school, elementary school.

**Participants:** this review will include youth and adolescent schoolgirls, university, or college female students.

**Interventions and setting:** The MHM interventions can be categorized as interventions involving menstrual education and/or the provision of menstrual supplies

- *Menstrual education interventions*: includes the provision of information, Health education, and training that are postulated to alleviate lack of MHM knowledge, attitude, and practice and associated psychological problem.
- *Menstrual supply interventions*: includes the provision of (1) Menstrual hygiene materials (products used to absorb menstrual flow, such as pads, cloths, tampons, or cups. (2)Menstrual supplies (other necessary kinds of stuff needed for menstrual hygiene that include soap and detergents, underwear, and anti-pain). 3) Menstrual facilities are those facilities that help to experience safe and dignified menstruation, such as toilets and water infrastructure and private space for washing, changing, drying, and disposing menstrual materials.([31](#_ENREF_31), [32](#_ENREF_32)).

**Comparator:** those who did not receive any of the aforementioned interventions**.**

**Outcomes:**  school attendance, school performance and school dropout, emotional wellbeing, menstrual knowledge, menstrual attitudes and menstrual hygiene practice. Because of the paucity of standardized measurements of the outcomes indicated we will include all studies that measured the outcome interest.

**Type of study design:** studies implemented randomized controlled trials, quasi-experimental or non-randomized trials will be included in this review.

**Publication Type**: peer-reviewed journal article, grey literatures, and dissertation will be included

**Exclusion criteria**: Reviewers will exclude studies not available in English, and conference abstracts will be also excluded.

## Source Information and Search strategy

Data will be searched using the following databases; PubMed, Google scholar databases udsing a mix of medical subject headings (MeSH) and relevant keywords related to menstrual hygiene management (MHM) interventions. Besides, grey literatures will be searched and citation lists will be used as an additional searching mechanism. Hand searches of key journals like the journal of adolescent health will be also conducted, from the start of indexing until the last date of searching. We will restrict the language to be English language. Search will be re-run just before the final analysis and further studies retrieved for inclusion. If the required literature is lacking important information needed for the review, we will contact the corresponding author.

| **Search #1:** “adolescent girls” OR “college students” OR “university student” OR “school girls” OR youth OR ladies OR puberty OR feminine OR gender OR parents OR mothers OR fathers OR community |
| --- |
| **Search # 2:** hygiene OR sanitizer OR sanitary OR sanitation OR washing OR soap OR “menstrual cup” OR “menstrual tampon” OR napkin OR pad OR products OR technology OR training OR “latrine access” OR toilet OR bathroom OR “menstrual hygiene” OR “Personal hygiene” OR “sanitation facilities” OR WASH OR “water supply” OR “water access” OR “water source” OR absorb OR absorbent OR “health education” OR “menstrual management” OR intervention |
| **Search #3:** “control group” OR homemade OR “worn out” OR rag OR cloth |
| **Search#4:** catamenia OR menarche OR menstruation OR menses OR “menstrual blood” OR “menstrual flow” OR “menstrual fluid” OR “menstrual period” |
| **Search#5:**  absenteeism OR absent OR “academic performance” OR “school attainment” OR “school attendance” OR “school dropout” OR “school missing” OR “academic failure” OR vocation OR distract OR anxiety OR shame OR ashamed OR bullying OR mock OR embarrassment OR fear OR fearful OR distress” OR “isolation” OR “harassment” OR “intimidation” OR “confused” OR “depress OR confidence OR empower OR “menstrual health” OR “menstrual knowledge” OR “menstrual attitude” OR “menstrual practice” OR “movement restriction” OR “quality of life” OR wellbeing OR “reproductive health” OR psychology OR “mental health” OR psychosocial OR secrecy OR “self-esteem” OR shame OR empower OR understanding OR worries OR worry OR upset OR infection |
| **Search # 6:** Search #1 and Search #2 and Search #3 and Search #4 and Search #5  Filters used: English language, Human |

Table-1: PubMed Search Strategy

## Data management and selection process

Identified, eligible studies will be imported into EndNote version X5 specific software for managing bibliographies. Two independent authors will share the EndNote to double-check the title and abstracts against the eligibility criteria. Studies that the two authors agreed upon will be subjected to full-text review. Any dispute will be settled by a third author and consensus will be sought by discussion. Then, full-text articles of the potentially relevant studies will be screened for the final inclusion of the study in the review according to the eligibility criteria.

## Data extraction process

Data extraction spreadsheet will be prepared and accordingly, data will be extracted manually and the spreadsheet will be populated with the interest of variables pertaining to the research question. From each study the following data will be extracted; 1) Author name, 2) Year of publication, 3) Location, Study design, 4) Population, 5) Sample size, 6) Duration of intervention, 7) Outcome measurement time 8) Description of intervention 9) Mode of intervention.

## Risk of bias in individual studies

The retrieved articles will pass through the quality assessment process. Thus Studies will be assessed for potential risk of bias, using JBI assessment tool, independently by two authors. (<http://joannabriggs.org/research/critical-appraisal-tools.html>). If there appears a dispute between the two reviewers it will be resolved by discussing with a third reviewer.

## Data Synthesis

# Data extracted will be summarized using tables, and there will be a narrative synthesis of around the type of intervention, target population characteristic, type of outcome, and summery of intervention.

Previous reviews: A previous review was done in 2016 ([15](#_ENREF_15)) however, it was conducted one year after the advocacy of the "MHM in Ten" agenda ([21](#_ENREF_21)). In the meantime, there were interventional studies conducted since then. Therefore, these studies are not included in the previous review. Hence, the current systematic review is warranted. Moreover, it will be help full to design evidence-based MHM interventions and intervention modalities in schools and will also lay the ground for feature studies and policies on school-based MHM interventions.

# **Discussion**

MHM has been a bottleneck for girls in many aspects. It affects the physical, psychological, social health, and school attainment. Taking this into consideration, a global initiative named MHM in ten calls up for multi-sectoral collaboration to achieve dignified and healthy MHM among school girls (Unicef, 2014). This will also help to achieve some of the SDG's; good health and well-being, inclusive and equitable quality education, gender equality and women’s empowerment, clean water and sanitation, economic growth, productive employment and decent work for all (United Nations 2020). Hence, responsible sectors need update and systematically documented evidence to work on it.

This review will give a full picture about the effect of school-based interventions on MHM KAP wellbeing, school attendance, and performance, among school girls. A previous review was done in 2016 ((Hennegan and Montgomery, 2016), however, it was conducted one year after the advocacy of the "MHM in Ten" agenda ( Unicef, 2014). In the meantime, there were interventional studies conducted since then. Therefore, these studies are not included in the previous review. Hence, the current systematic review is warranted. Moreover, it will be help full to design evidence-based MHM interventions and intervention modalities in schools and will also lay the ground for feature studies and policies on school-based MHM interventions.

# **References**

# 1. Consultation on Draft Long List of Goal, Target and Indicator Options for Future Global Monitoring of Water, Sanitation and Hygiene. WHO/UNICEF Joint Monitoring Programme (JMP) for Water Supply and Sanitation. 2012.

# 2. Sommer M, Chandraratna S, Cavill S, Mahon T, Phillips-Howard P. Managing menstruation in the workplace: an overlooked issue in low-and middle-income countries. International Journal for Equity in Health. 2016;15(1):1-5.

# 3. ItsTime For Action: Investing in Menstrual Hygiene Management is to Invest in Human Capital 2019 [cited 2020 June 29].

# 4. Changing perceptions around menstrual hygiene management and why it's important 2018 [cited 2020 June 29

# ].

# 5. Rasheed TO, Afolabi WA. Maternal and Adolescent Factors Associated with Menstrual Hygiene of Girls in Senior Secondary Schools in Lagos, Nigeria. Journal of Maternal and Child Health. 2021;6(1):35-45.

# 6. Girls in Control:Compiled Findings from Studies on Menstrual Hygiene Management of Schoolgirls. 2014.

# 7. Afiaz A, Biswas RK. Awareness on menstrual hygiene management in Bangladesh and the possibilities of media interventions: using a nationwide cross-sectional survey. BMJ open. 2021;11(4):e042134.

# 8. Phillips-Howard PA, Caruso B, Torondel B, Zulaika G, Sahin M, Sommer M. Menstrual hygiene management among adolescent schoolgirls in low-and middle-income countries: research priorities. Global health action. 2016;9(1):33032.

# 9. Sommer M, Schmitt ML, Ogello T, Mathenge P, Mark M, Clatworthy D, et al. Pilot testing and evaluation of a toolkit for menstrual hygiene management in emergencies in three refugee camps in Northwest Tanzania. Journal of International Humanitarian Action. 2018;3(1):1-14.

# 10. Shallo SA, Willi W, Abubeker A. Factors Affecting Menstrual Hygiene Management Practice Among School Adolescents in Ambo, Western Ethiopia, 2018: A Cross-Sectional Mixed-Method Study. Risk Management and Healthcare Policy. 2020;13:1579.

# 11. Sivakami M, van Eijk AM, Thakur H, Kakade N, Patil C, Shinde S, et al. Effect of menstruation on girls and their schooling, and facilitators of menstrual hygiene management in schools: surveys in government schools in three states in India, 2015. Journal of global health. 2019;9(1).

# 12. Chandra-Mouli V, Patel SV. Mapping the knowledge and understanding of menarche, menstrual hygiene and menstrual health among adolescent girls in low- and middle-income countries. Reproductive health. 2017;14(1).

# 13. Tegegne TK, Sisay MM. Menstrual hygiene management and school absenteeism among female adolescent students in Northeast Ethiopia. BMC public health. 2014;14(1):1118.

# 14. Sharma A, Kaur J. Artificial Intelligence Based System: Improving the Women Menstrual Hygiene. Information Resources Management Journal (IRMJ). 2021;34(2):80-90.

# 15. Hennegan J, Montgomery P. Do Menstrual Hygiene Management Interventions Improve Education and Psychosocial Outcomes for Women and Girls in Low and Middle Income Countries? A Systematic Review. PloS one. 2016;11(2):e0146985. Epub 2016/02/11.

# 16. Sumpter C, Torondel B. A systematic review of the health and social effects of menstrual hygiene management. PloS one. 2013;8(4):e62004. Epub 2013/05/03.

# 17. Ssewanyana D, Bitanihirwe BKY. Menstrual hygiene management among adolescent girls in sub-Saharan Africa. Global health promotion. 2019;26(1):105-8. Epub 2017/05/10.

# 18. Nabwera HM, Shah V, Neville R, Sosseh F, Saidykhan M, Faal F, et al. Menstrual hygiene management practices and associated health outcomes among school-going adolescents in rural Gambia. PloS one. 2021;16(2):e0247554.

# 19. Paudel R, Budathoki CB, Shahi P. Menstrual Restriction on Health, Education, and Empowerment: A Review of Literature. Europasian Journal of Medical Sciences. 2021;3(1):93-7.

# 20. Guide to menstrual-hygiene-materials [database on the Internet]. UNICEF. 2019 [cited 2019]. Available from: https://[www.unicef.org/media/91346/file/UNICEF-Guide-menstrual-hygiene-materials-2019.pdf](http://www.unicef.org/media/91346/file/UNICEF-Guide-menstrual-hygiene-materials-2019.pdf).

# 21. ‘MHM in Ten’: Advancing the MHM Agenda in WASH in Schools. 2014.

# 22. The Sustainable Development Goals Report 2020.

# 23. Federal Democratic Republic Of Ethiopia Ministry of Health Menstrual Hygiene Management in Ethiopia An Intersectional issue: Policy and Implementation Guideline Addis Ababa: 2016.

# 24. Oster E, Thornton R. Menstruation, Sanitary Products and School Attendance: Evidence from a Randomized Evaluation. 2010.

# 25. Fakhri M, Hamzehgardeshi Z, Golchin NAH, Komili A. Promoting menstrual health among Persian adolescent girls from low socioeconomic backgrounds: a quasi-experimental study. BMC public health. 2012;12(1):193.

# 26. Montgomery P, Ryus CR, Dolan CS, Dopson S, Scott LM. Sanitary pad interventions for girls' education in Ghana: a pilot study. PloS one. 2012;7(10):e48274. Epub 2012/11/03.

# 27. Fetohy EM. Impact of a health education program for secondary school Saudi girls about menstruation at Riyadh city. J Egypt Public Health Assoc. 2007;82(1-2):105-26.

# 28. Sommer M, Caruso BA, Sahin M, Calderon T, Cavill S, Mahon T, et al. A Time for Global Action: Addressing Girls' Menstrual Hygiene Management Needs in Schools. PLoS medicine. 2016;13(2):e1001962. Epub 2016/02/26.

# 29. Moher D, Shamseer L, Clarke M, Ghersi D, Liberati A, Petticrew M, et al. Preferred reporting items for systematic review and meta-analysis protocols (PRISMA-P) 2015 statement. Systematic reviews. 2015;4:1. Epub 2015/01/03.

# 30. Bettany-Saltikov J. How To Do A Systematic Literature Review In Nursing a Step-By-Step Guide-McGraw-Hill Education New York: Open University Press; 2012

# 31. Guide to menstrual hygiene materials. 2019.

# 32. VanLeeuwen C, Torondel B. Improving menstrual hygiene management in emergency contexts: literature review of current perspectives. International journal of women's health. 2018;10:169.
