# Supplementary material for: Menstrual hygiene management interventions and their effects on schoolgirls’ menstrual hygiene experiences in low and middle countries: A systematic review
Source: PLoS One. 2024 Aug 22;19(8):e0302523. doi: 10.1371/journal.pone.0302523 (PMC11340951; doi:10.1371/journal.pone.0302523)
Supplement: S1 Data — (DOCX) [file pone.0302523.s004.docx]

| **Author name and year and location** | **Study design** | **Population (P)and Sample size (SS)** | **Duration of intervention (DOI)**  **and Outcome measurement time (OMT)** | **Intervention Description (DI) and mode of intervention (MOI)** | **Outcome of interest** |
| --- | --- | --- | --- | --- | --- |
|  |  | **P:**  **SS:** | **DOI:**  **OMT:** | **ID**  **MOI**: |  |
|  |  |  |  |  |  |
|  |  |  |  |  |  |
|  |  |  |  |  |  |
|  |  |  |  |  |  |
|  |  |  |  |  |  |
|  |  |  |  |  |  |
|  |  |  |  |  |  |
|  |  |  |  |  |  |
|  |  |  |  |  |  |
|  |  |  |  |  |  |
|  |  |  |  |  |  |
|  |  |  |  |  |  |
