# Supplementary material for: Menstrual hygiene management interventions and their effects on schoolgirls’ menstrual hygiene experiences in low and middle countries: A systematic review
Source: PLoS One. 2024 Aug 22;19(8):e0302523. doi: 10.1371/journal.pone.0302523 (PMC11340951; doi:10.1371/journal.pone.0302523)
Supplement: S2 Data — (DOCX) [file pone.0302523.s005.docx]

**Table-2: Characteristics, Population, Intervention and Outcome of Studies Included in the Review**

| **Author name and year and location** | **Study design** | **Population (P)and Sample size (SS)** | **Duration of intervention (DOI)**  **and Outcome measurement time (OMT)** | **Intervention Description (DI) and mode of intervention (MOI)** | **Outcome of interest** |
| --- | --- | --- | --- | --- | --- |
| Abedian et al. 2011  Mashhad, Iran ([41](#_ENREF_41)) | Randomized controlled trial | **P:** 19–25-year-old Dysmenorrheic University girls  **SS:**  Planed SS**:** 209  Actual SS: 165 (Peer-led education group n=54; Health provider-led education group n=50; Control group n=61) | **DOI:** At base line and at two consecutive menstrual cycles (approximated to two months)  **OMT:** Immediately after intervention | **ID**: self-care education  Arm 1: received health provider-led self-care education  Arm 2: received peer-led self-care education  **MOI**: Small group discussions about self-care education held by health providers and peer educators | - Mean score of menstrual knowledge significantly increased in both groups compared to the control group (peer-led self-care group increased by 2.1 times and health-provider 2.5 times) - Negative concepts of mean menstrual attitude decreased in the peer-led self-care education group (56.6 vs. 40.2, p=0.009) more than the health-provider-led self-care education group (56.9 vs. 48.3, p=0.035). - Severity of dysmenorrhea decreased between the intervention arms and control arm but not significant between the intervention groups |
| Agbede et al.  2021 Ogun State, Nigeria ([42](#_ENREF_42)) | Quasi-experimental | **P:** 10–19-year-old rural school adolescent girls  **SS**: 120  (30 in each 4 study arms) | **DOI:**  4 weeks (number and length of sessions not indicated)  **OMT:** Immediately post intervention (at 4 week) and 6 weeks follow up | **ID:**  Health education related to menstrual hygiene practice   - Arm 1: peer-led education intervention - Arm 2: parent-led intervention - Arm 3: combination of both - Arm 4: Placebo | - Menstrual hygiene practice of the three intervention arms have significantly improved both at 4^th^ (immediate post intervention) and at 6^th^ weeks follow up. - While the thirds arm (combination of peer and parent recorded the highest mean score of practice |
| Austrian et al. 2019 Kenya ([43](#_ENREF_43)) | Cluster-randomized controlled trial  (With Four arms) | **P:** 10–21-year-old girls  **SS**: 3,276 schoolgirls | **DOI**: 25 sessions each lasting for 65-95 minutes for 18 months  (Weekly in 2017 and every two weeks in 2018)  **OMT:** after 18 months (Immediately after completion of intervention) | **ID:**  Arm 1: No intervention  Arm 2: Disposable sanitary pad  Arm 3: Reproductive health education  Arm 4: sanitary pad and reproductive health education  The sanitary pad and education include: one pack of Nia Teen disposable sanitary pads distributed monthly with pairs of underwear provided once per term  The reproductive health education includes puberty, gender, gender, power, and rights, being true to yourself  **MOI**:  Trained facilitators provided facilitated health education (FHE) and distribution of health magazine developed by ZanaAfrica based on the UNESCO International Technical Guidance on Sexuality Education incorporating gender and power in sexuality and HIV education | - Provision of Pads improved menstrual hygiene management - RH education led to improved SRH knowledge, self-efficacy, gender norms and attitudes on menstruation - Combined intervention had stronger impacts on reducing shame/stigma around menstruation - None of the interventions had an impact on education outcomes like school attendance and enrolment for the subsequent grade |
| Babapour et al. 2022  Sari, northern Iran ([44](#_ENREF_44)) | Quasi-experimental non-randomized controlled trial | **P:** 11th-grade single students with regular menstruation  **SS: 90**  (30 in each of the three arms) | **DOI:** Six, one hr. sessions twice a week in WhatsApp messenger.  **OMT:** Not indicated | **ID**: The education sessions included: menstruation and menstrual disorder including PMS and measures to alleviate, life skill, female reproductive system   - Arm 1: received education by peer - Arm 2: received education by a healthcare provider - Arm 3:is control group   **MOI**   - Education held using WhatsApp messenger - All three groups received routine school counseling. - Education providers individually uploaded the pre-prepared audio files with the related PowerPoint file in each session & allowed participants to ask questions. At the end of each session, the healthcare provider/peer asked questions about the topics and motivated to participate in the discussion. | **Primary outcome:** Premenstrual syndrome (PMS)   - PMS score decreased in the intervention groups compared to the control group. - The effect size in the education by a health care provider group (Partial Eta Squared = 0.82, p < 0.0001) was more than the education by peers’ group (Partial Eta Squared = 0.67, p < 0.0001).   **Secondary outcomes**: General health and premenstrual dysphoric disorder   - The mean score of general health (measure of emotional distress) significantly decreased in the education group by peers (Cohen's d = 0.25, p<0.0001) and education by health care provider group Cohen’sd=0.37, p<0.0001) compared to control group. - Intervention did not significantly reduce the frequency of premenstrual dysphoric disorder among the two intervention groups as compared to the control group (p>0.050). |
| Belay et al.  2020 Tigray Ethiopia ([35](#_ENREF_35)) | Quasi-experimental | **P:** Grade 7–12 students  **SS:** 8,839 Students in 15 intervention schools | **DOI**: one academic year  **MOT:** immediately post intervention | **ID:**  Menstrual education provided to boys and girls   - Girls provided with menstrual hygiene kits containing four locally produced, reusable menstrual pads and two pairs of underwear.   **MOI**: School-based distribution of a booklet called Growth and Changes, written in English and Tigrinya (the local language).   - Students encouraged to take the booklet home with them to share with their families. - Additional oral instruction was provided on-site by project staff from Mekelle University - Interactive question and answer sessions - Distribution of 12 211 pamphlets - Distribution of menstrual kit - Demonstration of how to use the sanitary pads for girls | Girls had 24% fewer absences as compared to the control arm during the post-intervention period. |
| Blake et al. 2017; Oromia Ethiopia ([45](#_ENREF_45)) | Cluster-randomized study  triangulated with qualitative approach | **P:** Grade 6 &7 schoolgirls  **SS:** 636 | **DOI:** Puberty book provided to the girls for 4 weeks  **OMT:** Four weeks after the distribution of book (no follow up in between) | **ID:** The Ethiopia version of the girl’s puberty book Growth and Changes.  The book aimed at girls aged 10 to 14 years, covering puberty education, menstruation and menstrual hygiene management; and culturally tailored stories.  **MOI:** Book delivered to the study participants to read them. | Intervention had positive effect on:   - The girls’ knowledge about menstruation with effect size of 0.6 (medium effect size - Post intervention, girls in the intervention group were less likely to indicate that they felt fear regarding menstruation (OR = 0.70, 95% CI = [0.51, 0.95]) or shame (OR = 0.61, 95% CI = [0.38, 0.96]) than girls in the control group. |
| Fakhri et al. 2013  Mazandaran province, Iran ([31](#_ENREF_31)) | Quasi- experimental  (Non-randomized controlled cluster trial | **P:** 14 -18-year-old-girls with  low socio-economic status from  urban and rural public high schools  **SS:** 689 (349 intervention group and 349 control group) | **DOI**: (20 hrs.) 10 sessions of 2 hr. each  (Not indicated for how long)  **OMT:** At the end of the education intervention | **ID:** Training about:   - personal health and hygiene during Menstruation - Significance of adolescence, physical and emotional changes during adolescence, - Pubertal and menstruation health and premenstrual syndrome   **MOD**: intervention provided by Youth and School Health department to the intervention arm | - **Menstrual health** especially bathing and genital hygiene improved (61.6% in the experimental group compared with 49.3% in the control group engaged in usual bathing during menstruation (p = 0.002)) - **Attitude towards menstruation** was also significantly related to menstrual health. |
| Nyadoy et al. 2022 Uganda ([46](#_ENREF_46)) | Randomized Controlled Trial | **P:** primary school adolescent girls who reached menarche  **SS:** 60 (30 control and 30 intervention group | **DOI:**  one hour session twice a week, after classes, for period of six weeks  **OMT:** Outcome assessed immediately after the intervention ended | **ID:** Menstrual health management storying and gamification  **MOI**: Storying involved Senior Women Teachers and other invited role models sharing stories about the facts and myths of menstruation and menstrual hygiene management. The games involved competitive ball games such as soccer, netball, and rope work | - Girls in the treatment group (t = 8.498, df = 29, p < .05) obtained significantly higher scores (in four courses, English language, Mathematics, Integrated Science, and Social Studies) than those in the control group - Experiment group reported positive attitudes and expressed feelings of liberation from fear of boys during menstruation, |
| Oster et al. 2011  Chitwan District, Nepal ([34](#_ENREF_34)) | Randomized controlled trial | **P:** Grade 7 and 8 schoolgirls  (25 girls assigned to treatment group from each school)  **SS:** 198 | **DOI**: One school year intervention  **OMT:** Outcome assessed immediately after the intervention | **ID:** Menstrual cup branded as Moon-cup  **MOI:** Treatment girls and their mothers provided with menstrual cup and instructions on how to use it.  Girls provided with booklet of time diaries that included a menstrual calendar on which they were to note the start and end date of their period in each month. | - The menstrual cup does not significantly increase school attendance |
| Paul Montgomery 2012  Ghana ([32](#_ENREF_32)) | Non-randomized- controlled trial | **P:**12 - 18-year-old schoolgirls  **SS:** 120 | **DOI:**  Five months  **OMT**: At third and fifth month (at the end of the intervention) | **ID:**  Provision of one pair of underwear and twelve pads per month for the duration of the study with instruction and demonstrations on how to use and dispose of the sanitary pads.  Puberty educational about development of secondary sex characteristics, menstruation, and pregnancy, hygiene and menses management  Arm-1: Pads + puberty education  Arm-2: Puberty education  Arm-3: Control  **MOI:**   - Trained research assistants provided the puberty education - All participants received a daily calendar, pencil and sharpener to record their menstrual cycles | **Arm-1** (pad + education): school attendance improved significantly among participants, (lambda 0.824, F = 3.760, p, .001)  **Arm-2:** education only resulting in a similar school attendance level (M = 91.26, SD = 7.82) all of which were higher than control (M = 84.48, SD= 12.39). The effect size, partial eta-squared, was 0.094. |
| Paul Montgomery et al. 2016  Uganda ([47](#_ENREF_47)) | Cluster Quasi-Randomized Controlled Trial | **P:** Grade 3-5 schoolgirls    **SS:** 356 pre and post menarcheal girls) from 8 rural schools | **DOI:** Single session of puberty education and two times of pad distribution and soap (one sachet, 45gram (18 months apart)  The education session last for 1.25hrs  **OMT**: two years later | **ID:**  provision of reusable pad and Puberty education about menstruation, early pregnancy, life skills, prevention of HIV, strategies for avoiding sexual assault, healthy relationships, and friendship formation and goal setting.  **Arm-1:** puberty education  **Arm-2:** provided with reusable pad 3 pairs of underwear, one sachet, and 45 grams of soap with which to wash the pads.  **Arm-3:** puberty education and reusable sanitary pad  **Arm-4:** A control condition | - Control schools had 17.1% (95%CI: 8.7– 25.5) greater drop in school attendance than those in any intervention school - No psychosocial change was observed among study arms |
| Phillips-Howard et al. 2016  Gem  District Kenya ([48](#_ENREF_48)) | Cluster randomized  controlled feasibility study  open-level RCT | **P**: 14–16 years old girls (with no precluding disability) who experience at least three menses  **SS**:  **Planed SS:** 3165  **Executed SS:** (644 analyzed) from  30 rural primary schools | **DOI:** 15 months  **OMT:** at the end of the follow up (intervention) | **ID:** Girls in all arms received puberty and hygiene training; hand-washing soap; and pencils for calendar completion.  **Arm 1:** received one menstrual cup with written and verbal instruction of how to insert and clean  **Arm 2: r**eceived 16 disposable pads and relevant instruction.  **Arm 3:** Control  **MOI**: Nurses provided menstrual product specific training from study nurses after enrolment | - **School dropout (primary outcome):** Cups or pads did not reduce school dropout (control=8.0%, cups=11.2%, pads=10.2%) - **Absence:** Could not be analyzed because self-reported school absence was very rarely reported. - **STI (secondary outcome)**: Lowered prevalence of C. trachomatis and T. vaginalis but not N. gonorrhea. Greatest impact was among girls who had been exposed to intervention for at least 9 months or 12 months. - Prevalence of all STIs at the end line survey was 7.7% in the control arm versus 4.3% in the pooled cups +pads arms - **RTI**: Bacterial vaginosis was lower among cup arm (not significant), but not in the pad arm. - **TSS**: No case reported |
| Rezaei,et al. 2022  Iran ([41](#_ENREF_41)) | Quasi-experimental study | **P:** 13 - 16- year-old high school students and their mothers  **SS:**  **Control**: 111 (56 student and 55 mothers)  **Intervention**: 112 (58 student and 57 mothers) | **DOI:**  Not indicated  **OMT**: Immediately after intervention and three months latter | **ID:** Educational intervention based on the PRECEDE model provided.  Adolescence, puberty, menstrual cycle, abnormal signs, and common problems associated with menstruation, menstrual health, exercise, nutrition, mobility, and pain control in menstruation  **MOI:** The education was provided in 3 sessions of two hour each using lecture, face-to-face discussion, and question/answer methods for students and mothers in the intervention arm | - The mean score of menstrual health behavior was significantly higher in the intervention group than in the control group, immediately (P < 0.001), and three months after intervention (P = 0.02) - Mothers’ knowledge, attitude, and practice regarding menstrual health behaviors was significant reinforcing factor among intervention group compared to control group |
| Setyowati et al.2019  Indonesia ([49](#_ENREF_49)) | quasi-experimental  pre–posttest with a control group design | **P:** 9-12 years old schoolgirls how had not yet experienced menarche  **SS:** 174 girls | **DOI**: Not indicated  **OMT:** Not indicated | **ID:** Booklet containing information about preparation for menarche, reproductive organ, physical changes during adolescence, problems during menstruation and how to deal with it and menstrual hygiene  **MOI**:  Distribution of booklet to the intervention group | - Increased menstrual knowledge (OR = 45.1; 95% CI: 13.8–148.1) - Positive emotional response (OR = 12.7; 95% CI: 5.6–28.5) - Positive attitude towards menstruation (OR = 12.4; 95% CI: 5.8–26.6) |
| Sol et al. 2017 Bangladesh ([50](#_ENREF_50)) | Cluster randomized impact evaluation | **P:**  junior secondary school girls  **SS:** planned SS**:** 3862 girls  Actual SS: 2127  (595 treatment-1, 570, treatment-2 and 962 control group)  4,500 mothers/guardians and 4,500 fathers/guardian attended the Household education sessions | **DOI:**  least twice a month form 2017-2019  **OMT:** two years ( at the completion of the intervention ) | **ID:**  Construction and maintenance of menstrual health-friendly toilet facilities at school.  Incorporating puberty- and MH-modules in the school curriculum,  2-day session to increase menstrual health knowledge and understanding of the benefits of safe menstrual hygiene was produced to parents /guardians  **Arm 1:**  schools receiving a school program  **Arm 2:** schools receiving a school program combined with a targeted household program (‘combined program’)  **Arm 3:** control schools  **MOI:**   - Extensive campaign to familiarize teachers, students and parents, next to festivities, Group discussions, essay writing competitions and screening of a tv-show and extracurricular activities | **Primary outcome:** educational outcomes, psychosocial outcomes and empowerment of adolescent girls.   - Absence rates in treatment schools are significantly lower than in the control schools (no significant difference between the school program and combined program schools) - School dropout reduced in both treatment group as compared to controls   **Secondary outcomes**:   - Increase in the knowledge of girls about menstruation and menstrual health (on both treatment arms - Lowered restrictive beliefs surrounding the mobility of girls on their menses. (On both treatment arms) - More likely to get permission to go to the toilet when they asked their teacher - No treatment effects on teasing during menstruation |
| Wilson et al. 2014  Rural Kenya ([51](#_ENREF_51)) | Cluster randomized control | **P:** Schoolgirls  **SS:** 302  (143intervention ,159 control) | **DOI:** One session  **OMT**: One month after intervention | **ID:** Training on how to make a reusable sanitary pad and provision of equipment to make three reusable pads.   - Providing printed hand-out, as reminder on how to make the pad and instructions about washing and drying, risk of infection or irritation of damp or poorly washed pad; with suggested ways to dry the pad outside and avoid embarrassment. - Did not include general menstrual health education in order to evaluate the merely effect of pad use   **MOI**: training and provision of handout | - Mean number of days of school missed decreased or stayed constant among treatment group while schools in the control group either stayed constant or increased |
