# Supplementary material for: Menstrual hygiene management interventions and their effects on schoolgirls’ menstrual hygiene experiences in low and middle countries: A systematic review
Source: PLoS One. 2024 Aug 22;19(8):e0302523. doi: 10.1371/journal.pone.0302523 (PMC11340951; doi:10.1371/journal.pone.0302523)
Supplement: Attachment — Submitted filename: Response to Reviewers .docx [file pone.0302523.s006.docx]

**Comments to the Author**

1. Is the manuscript technically sound, and do the data support the conclusions?

The manuscript must describe a technically sound piece of scientific research with data that supports the conclusions. Experiments must have been conducted rigorously, with appropriate controls, replication, and sample sizes. The conclusions must be drawn appropriately based on the data presented.

Reviewer #1: Yes

Reviewer #2: Yes

2. Has the statistical analysis been performed appropriately and rigorously?

Reviewer #1: Yes

Reviewer #2: N/A

3. Have the authors made all data underlying the findings in their manuscript fully available?

The [PLOS Data policy](http://www.plosone.org/static/policies.action#sharing) requires authors to make all data underlying the findings described in their manuscript fully available without restriction, with rare exception (please refer to the Data Availability Statement in the manuscript PDF file). The data should be provided as part of the manuscript or its supporting information, or deposited to a public repository. For example, in addition to summary statistics, the data points behind means, medians and variance measures should be available. If there are restrictions on publicly sharing data—e.g. participant privacy or use of data from a third party—those must be specified.

Reviewer #1: Yes

Reviewer #2: Yes

4. Is the manuscript presented in an intelligible fashion and written in standard English?

PLOS ONE does not copyedit accepted manuscripts, so the language in submitted articles must be clear, correct, and unambiguous. Any typographical or grammatical errors should be corrected at revision, so please note any specific errors here.

Reviewer #1: Yes

Reviewer #2: Yes

5. Review Comments to the Author

Please use the space provided to explain your answers to the questions above. You may also include additional comments for the author, including concerns about dual publication, research ethics, or publication ethics. (Please upload your review as an attachment if it exceeds 20,000 characters)

Reviewer #1: I would encourage the authors to tighten the piece prior to publication in two ways. First, the nature of the search seems to have translated into several important studies escaping capture as part of the dataset. This is not fatal; it is the nature of the searches the authors chose to run. But I think the authors will want to cabin their conclusions as being only as good as the data the searches yielded, acknowledging that other work that did not fit the authors' relatively narrow criteria were therefore excluded. Second, I think the authors would be well-served to address the linguistic challenges in talking about "women and girls" and "menstrual hygiene," when those terms have been made much more complicated by excellent work in this field. The authors gesture at this by the use of the term "menstruand" (which I found jarring and out of synch with most other scholarship in the field," but they never address the issue head-on.

**Authors’ response**

- Dear reviewers, the conclusion provided in the manuscript is yielded from the findings (literatures reviewed). The findings evidenced that the interventions can enhance schoolgirls' educational outcomes, and can improve their menstrual knowledge, attitudes, and practices by helping them manage their periods more effectively.
- We used the term “Menstruant” not “menstruand” may be it is editorial error. The use of women and girl is more dominant in WASH sectors. While menstruants is emerging form gender inclusive perspective, hence, in the write up we tried to balance the use of the terms. However our search clearly identified literatures that assessed the effect of menstrual interventions on “school girls”.

Reviewer #2: General
Much is written in passive voice – active voice would be preferable. Occasionally you use ‘we’ – you should pick one narrative style and stick to it.

I don’t think it’s necessary to have the acronyms listed since there are only two (unless this is a requirement of the journal then please ignore me). You haven’t included PICOT.
There are times when you spell out MHM and others when you use the acronym – be consistent and use the acronym throughout
The paper should recognise that it isn’t just cisgender women who menstruate but potentially also transgender men, non-binary and other gender diverse persons. The term ‘menstruator’ could be used instead of women and girls to be inclusive.
I think it would be helpful if you stated the countries where studies present different evidence else you might fall into making sweeping generalising comments. For example lines 88-89.
You’ve used WASH as an acronym and spelled out but haven’t introduced it as an acronym – please revise
You could consider adding a positionality statement at the beginning of the paper.
Why were no studies in any Ethiopian languages selected?

**Authors’ response**

- We have converted passive voice to active voice in most of the relevant sections.
- Acronyms have been spelled out and made consistent in the current version.
- Although "Menstruants" is a gender inclusive term, the primary objective is to assess its effect on “school girls” and we had to be specific to these group of study participants. Moreover, the studies we have reviewed mostly used the term "Schoolgirls," "Girls," and "Women". For this reason, we maintained the terms. However, in the write up section we have also considered “menstruants”
- The findings are form different low and middle income countries, and citation is in place **( line 89)**
- The academic and research language for Ethiopia is English. So, there is no study conducted using Ethiopian language and which is not included.
- We have included positionality statement under the method section on the current version (**line 128)**

**Abstract**
‘intervention’ seems like a rather broad keyword

**Authors’ response**

**“**Intervention" is preceded by "menstrual hygiene management" and expressed as “menstrual hygiene management interventions” to be more specific, (lines 27 and 34)

MHM definition – could point out that depending on the materials used somewhere to wash and dry reusable materials is also necessary. Could also mention the access to and ability to wash and dry underwear is also a necessity.

**Authors’ Response:** Agreed, we utilized the standard definitions, and employing operational definitions may provide further assistance.

Lines 84-87 its clearer written as: More than half (52%) of adolescent girls in Ethiopia have never received any information about menstrual hygiene due to socio-cultural
87 misinformation, religious taboos, and inadequate menstrual supplies and facilities, which leads to fear, confusion, and lack of confidence when menarche occurs (11-15).
Line 93 – what is meant by gender empowerment exactly? Vague phrase it might be interesting to unpack it.
111-112 you use the word review a lot
Good justification for the work.

**Authors’ response**

The issue is fixed accordingly: **(line 86-89)**

- “More than half (52 %) of adolescent girls in Ethiopia have never received any information about menstrual hygiene (10), due to religious taboos, socio-cultural misinformation, and inadequate menstrual supplies and facilities, which leads to fear, confusion, and lack of confidence when menarche occurs (11-15).”
- “gender empowerment” modified to “gender equality” **( line 93)**

Methods
I wonder if it would produce, more results if you googled gender neutral terms like ‘menstruator’ – it could be written as a limitation if you didn’t do this.

**Authors’ response**

- In fact, we purposefully limited our search terms to be gender-specific to highlight the impact the interventions on schoolgirls, but it is still valued concern because it may have limited searching relevant literature. We have included it on the limitation section of the manuscript

The systematic review following PRISMA is clearly and transparently described. Having three reviewers to judge papers against the outlined eligibility criteria reduces bias.
Line 175 - I don’t think mode of intervention needs an explanation.
The search strategy is well documented and comprehensive
Data bases and other sources of information are specified
The process of study selection is clearly outlined.
The process of data extraction is well documented
The key characteristics of included studies is clearly presented.
The studies were rigorously assessed for risk of bias using the Joanna Briggs Institute critical appraisal assessment tools

**Authors’ Response**

Written as “Mode of intervention” only (**Line 200, in the modified version)**

Results
Line 192-193 - I think you mean Saudi Arabia, not Saudi Riyadh

**Authors’ Response**

- Saudi Arabiya is excluded from the analysis as it is one of the high income countries. And modification is made on the manuscript accordingly **( Line 215)**

From line 251 – have you listed all of the education components because saying they ‘included things such as…’ makes it seem that the list is not exhaustive, but it seems to be that way. And perhaps it should be if not too long to include each component.

**Authors’ Response**

Modified as “The menstrual education components of the studies included; puberty education, training on…” (**line 239)**
Line 279 - Good and transparent analysis of intervention fidelity
Interesting discussion on blinding.
Line 305 – et al is repeated

**Authors’ response:** Amendment made (the repeated et al is deleted/ **line 295**)

Table 2 and 3 – could we have color code for severity of bias?

**Authors’ response**: Color code is given accordingly

Table 4 – reduce space between lines so table is less spread out – its currently across 13 pages

**Authors’ response**: Resolved (line space of the table is reduced)
Table 4 – expand acronyms
Table 5 – I assume the 0 means no impact and + means positive impact but you need to add this key somewhere. It would be clearer if you also did a colour i.e. green for positive effect, yellow for neutral. It would also look better if the symbols were in the centre of the square. Table 5 – could you add the type of intervention into the summary table and group similar types of intervention together?

**Authors’ response**

- Acronyms are expand on the heading section
- Key is provided for Table-4 on **line 337**

Discussion
370 – was the not paying attention due to hormonal fluctuations or worry and concern about not being able to manage their period?

**Authors’ response**: lack of attention in education could be attributed by both the hormonal fluctuations and worry about not being able to manage their period. We have added additional literatures to illustrate it. **(Line 353)**

An important narrative I feel is missing more on which there is a growing body of literature on is the role of men/boys/non-menstruators is upholding stigma/teasing etc. you touch upon this in lines 405-406 but feel some more literature on how non-menstruators act as a barrier could be added in.

**Authors’ Response:** Noted and we have added more details as follows

“One article exploring the beliefs and attitudes boys and men hold about menstruation revealed that men generally have more negative attitudes towards menstruation and view it as debilitating and requiring secrecy. However, these attitudes may soften as men age and gain more knowledge and experience with menstruation” **(line 390)**

407 – careful not to generalize all religious beliefs be specific.

**Authors’ response:** Addressed in **line 394** as “most religious beliefs”, to avoid generalization.

Implications
447-449 – be more specific – state the unmet needs

**Authors’ Response:** specified on the current version on **line 435-439**

“Governments and policymakers should prioritize menstrual hygiene as a public health issue and develop policies and guidelines to meet the needs of menstruating girls including affordable menstrual products, proper hygiene facilities, education about menstruation, and access to healthcare services for managing menstrual health.”

Conclusion
Seems like the conclusion is the first instance apart from the title where you mention low- and middle-income countries… why would this not be an issue in high income countries? The United Kingdom is currently going through a cost-of-living crises where people are in hygiene poverty – unable to buy basic hygiene items like menstrual pads. Ref - <https://journals.plos.org/plosone/article?id=10.1371/journal.pone.0255001&utm_source=miragenews&utm_medium=miragenews&utm_campaign=news>
Furthermore – Saudi Arabia is one of the countries studied, which is a high income country.

Is categorizing countries by their economic status quite western and neoliberal?

**Authors’ response**

- Menstrual hygiene management is indeed an issue that affects individuals in both high-income and low-income countries. In high-income countries, access to menstrual products and facilities for proper hygiene may be more readily available (as compared to low income countries), but issues such as stigma and access to education about menstrual hygiene persist. In low-income countries, the challenges may include limited access to sanitary products, clean water, and sanitation facilities, as well as social stigma and inadequate education about menstrual health. Therefore, addressing menstrual hygiene management is an important aspect of promoting gender equality and ensuring the well-being of individuals across different socioeconomic contexts.
- We have used the World Bank classification of countries based on economic status and we have excluded the finding from Saudi Arabia.
- Categorization is made by income level ( world bank)

6. PLOS authors have the option to publish the peer review history of their article ([what does this mean?](https://journals.plos.org/plosone/s/editorial-and-peer-review-process#loc-peer-review-history)). If published, this will include your full peer review and any attached files.


If you choose “no”, your identity will remain anonymous but your review may still be made public.


**Do you want your identity to be public for this peer review?** For information about this choice, including consent withdrawal, please see our [Privacy Policy](https://www.plos.org/privacy-policy).

Reviewer #1: No

Reviewer #2: **Yes:** Georgia Hales
