# Supplementary material for: Menstrual hygiene management interventions and their effects on schoolgirls’ menstrual hygiene experiences in low and middle countries: A systematic review
Source: PLoS One. 2024 Aug 22;19(8):e0302523. doi: 10.1371/journal.pone.0302523 (PMC11340951; doi:10.1371/journal.pone.0302523)
Supplement: Attachment — Submitted filename: Response to reviweres.docx [file pone.0302523.s007.docx]

**Reviewer #**1:

**Line 24**: uses passive voice

**Authors ‘Response**

Dear reviewers I agree with comments in line #24

The statement in line# 24 is replaced with active voice, “To address these issues, researchers have conducted intervention studies, but the impact on school attendance has varied”

**Line 32:** “Accordingly” doesn’t flow from previous sentence
**Authors ‘Response**

I agree with comment of the reviewer, the statement in line 32 is paraphrased as

“Review of sixteen trial studies showed that menstrual hygiene interventions have a positive effect on schoolgirls' school attendance, performance, and dropout rates, as well as on their menstrual knowledge, attitudes, practices, and emotional well-being.”

**Line 35:** “However” is clunky here.

**Authors ‘Response**

The comment is well taken and “However” in line # 34 is omitted and the sentences is rephrased as “There was a low to medium risk of bias in the most of the studies.”

**Line 82:** You use the word “menstruants” without explaining this word choice. This absolutely needs to be addressed. It is not widely accepted (by two different “camps” for different reasons). It is jarring and merits context. If you believe that this is the term that is emerging, you should at least cite to some authority for that, because I don’t think there is consensus/agreement that many readers will have heard it before (“menstruator” is much more common in significant literature).

**Authors ‘Response**

I agree with point raised and it is valued concern. Hence the word “menstruants” in line 82 is replaced with “menstruators” as per the recommendation

**Lines 84-89:** You definitely need to explain your focus on women and girls. Your response to the reviewers makes clear that it is because that is what the studies you survey are studying…but this does not come through in your paper. The reader is still left wondering why you are focusing on *girls* (but then again, are you focused just on girls? In fact, later in the paper, such as at Lines 296-98, you do cite studies involving boys). This needs to be clarified.

**Authors ‘Response**

The comment is well accepted. The reason for focusing on schoolgirls is now explained in line 419-426 as follows, to make it clear to the reader.

**“Most of the literature about menstrual hygiene management, especially in low- and middle-income counties, don’t adequately address the needs of people who identify as gender-nonconforming.** Menstrual discourses, are frequently written from the perspective of a cis woman, highlighting only the menstrual experiences of adolescent girls and cis women. **This exclusion may affect menstrual hygiene needs and experiences of t**ransgender, non-binary, and other gender varied individuals”

This review investigated how involving males (boys, fathers, or parents) in interventions might improve schoolgirls' experiences. We included intervention studies targeting these male groups alongside traditional MHM interventions. This broadens the range of interventions studied, but the outcome/impact must be measured specifically on schoolgirls.

**Lines 95-96**: I don’t think the average reader will understand what you mean by “weak enabling environments”’

**Authors ‘Response**

The comment is well accepted. In the old version we explained the word “weak enabling environments”’ using the list of challenges that came after it . i.e. “Insufficient knowledge about menstruation, inadequate access to water, sanitation and hygiene services, lack of adequate hygiene materials, and social norms unsupportive of those who menstruate”

But if it creates confusion for readers, the already listed challenges are more descriptive and we have omitted the phrase “weak enabling environment” to avoid ambiguity to the reader

**Line 111**: comma missing

**Authors ‘Response** .

This is well taken and Comma is used in line #110 “2015-2016 school year, demonstrated”

**Line 114:** reviews “were conducted” (passive voice) by WHOM?

**Authors ‘Response**

Dear reviewer, we have included the list of 4 citations which helps to address the potential question about “who conducted the review?”. The 4 citations in line 116 can address this concern than listing the name of the authors who did the review.

**Lines 143-145**: Here you talk about limiting your search to studies in which the participants were schoolgirls only. Is this accurate? See comment above re Lined 296-98. Perhaps I am misunderstanding.

**Authors ‘Response**

Dear reviewer, your understanding is correct. The statement is rephrased to “

The search was limited to studies that measured outcomes on schoolgirls because the objective of the review was to evaluate how menstrual hygiene management intervention programs impact schoolgirls' attendance, academic performance, or dropout rates. ” The study aimed to evaluate the effects of intervention programs on school attendance, performance, and dropout rates of schoolgirls, as clearly stated in lines 144-145. Therefore, while the intervention may involve parents, the community, and males, the outcomes must be assessed specifically for schoolgirls. We have included studies that involved parents or males in the intervention, with the actual outcomes measured for the schoolgirls. Additionally, we used terms such as fathers, mothers, community, and parents as search terms to encompass various types of interventions.

**Line 148**: Punctuation missing?

**Authors ‘Response**

This is well noted punctuation is in place now, “supplies**.**”

**Lines 149, 152, 160**: Colon not necessary?

**Authors ‘Response**

Well noted and accepted, the colon in line 149 and 152 is removed and we kept the colon in line 159 and we deleted the word “includes” to make appropriate use of colon

**Line 165**: Unnecessary comma
**Authors ‘Response**

Well accepted, comma in line 165 is removed the statement is written as “we excluded studies not available in the English language and conference abstracts.”

**Line 210**: Do you mean “findings” (plural)?

**Authors ‘Response**

This is noted, line 208 in this version, is changed into plural “a summary of the findings”

**Line 212**: Are “menstrual interventions” the same as “menstrual hygiene management interventions” discussed on Line 146?

**Authors ‘Response**

Yes, it is the same and line 210 is corrected as “Sixteen trial studies that assessed the effect of menstrual hygiene management interventions…”

**Line 254**: Passive voice
**Authors ‘Response**

Dear reviewer the comment is accepted, the statement in line 252 is changed into active voice as follows

“Montgomery et al. suggested that using school attendance and dropout rates as a proxy indicator of academic performance”

Line 258: Odd initial cap for “School”

**Authors ‘Response**

This is accepted. Line 256 Changed from Official School attendance🡺 Official school attendance

Lines 272-73: Awkward and difficult to follow sentence.

**Authors ‘Response**

The comment is well accepted. The statement in line 270-272 which was “Montgomery et al. conducted study on comparable peri-urban schools but included one remote rural site without experience in using sanitary pads that had no electricity, and no paved roads.”is rephrased as follows:

“Montgomery et al. carried out study on peri-urban schools that were comparable, but they also incorporated a remote rural site lacking experience in using sanitary pads, with no access to electricity and unpaved roads.”

Line 298: Were these period underwear or regular underwear?

**Authors ‘Response**

Dear reviewer, fortunately I physically knew the underwear’s provided in this specific intervention and they are normal/regular underwear’s and the term “ regular” is added ( line 297-298)

**Lines 300-302**: Run-on sentence.

**Authors ‘Response**

Well noted and accepted. The statement in line 297-299 “School attendance (data collected prospectively on-site by team researchers) was analyzed before and after the intervention and also compared with attendance data from the prior school year”

Is amended as

“School attendance was analyzed before and after the intervention and also compared with attendance data from the prior school year”

**Line 464**: Instead of talking about “menstruating girls and women,” might this be an appropriate place to talk about “all who menstruate,” notwithstanding your focus

**Authors ‘Response**

Dear reviewer this comment is accepted. “Menstruating girls and women “is replaced with “all who menstruate” on the current version ( line # 470)
**Line 466**: Queer theory would ask us to look rigorously at the word “normal.” I think you mean “involuntary” or “inevitable” (or perhaps drop the adjective entirely) (line # 474).

**Authors ‘Response**

Well Noted, the word “Normal” is dropped from line # 471

**Reviewer #2: Dear authors**,

Thanks for the effort you’ve put in to addressing my comments. I feel like the paper has now avoided a couple of easy pitfalls. The information in the tables is also now much easier for the reader to digest – thanks for this. There are just a few things I’m not sure were fully addressed; perhaps I did not explain myself very well so here I elucidate:
I understand the want to just focus on one particular group (schoolgirls). However, I raised this point as it is often gender diverse persons who are left out and not represented in this type of data collection, which is something that needs to change if the research is to remain relevant going forwards. I appreciate the mention of menstruators – it’s important as a limitation if anything to explain the data set is solely focused on cisgender girls and women.

**Authors ‘Response**

The following paragraph would explain the limitation, which is placed in line # 416-421

**“****Most of the literature about menstrual hygiene management, especially in low- and middle-income counties, don’t adequately address the needs of people who identify as gender-nonconforming.** Menstrual hygiene issues, are frequently written from the perspective of a cis woman, highlighting only the menstrual experiences of adolescent girls and cis women**. This exclusion may affect menstrual hygiene needs and experiences of t**ransgender, non-binary, and other gender varied individuals.”

Largely you give the country where the reference is from but not all the time. It’s important to do this consistently in order to avoid making generalization about all low-income countries.

**Authors ‘Response**

The input is considered and included the countries where the citation exists, but country was not included mainly on table 5

I’m happy to see the inclusion of the positionality statement – it adds transparency. Something I was looking for was mention of the country/countries the authors are from. There adds incongruence if the authors were from a high-income country say but are commenting on low income countries. How does where the authors are from impact the direction of the study or interpretation of results?

**Authors ‘Response**

Dear reviewer this is well accepted. The following statement is added the statement “I am from Ethiopia one of the low-income countries” in line 130 to indicate the principal investigator is from low income country.

That’s a shame that you’ve now had to exclude Saudi Arabia. I hope my point that menstrual health is a global issue that impacts high-income countries was taken not to diminish the lack of access in lower income countries but to highlight it’s not just an issue in low-income countries and that there are inequalities within countries as well as between them.

**Authors ‘Response**

Dear reviewer, issues of menstrual poverty and inequity are global challenges. It is widely recognized that individuals who menstruate in high-income countries encounter various challenges, although the severity and nature of these challenges may vary from LMIC. Our initial focus for the review was on addressing issues in low and middle-income countries. And we wrongly classified Saudi Arabia as LMIC and include it in the review process. However, this does not imply that menstrual hygiene management is not an issue in high income countries. Your feedback in the previous review highlighted the inclusion of one high-income country, Saudi Arabia, in the review. This was an important observation. It would not be appropriate to include a high-income country when the review is intended to focus on LMICs. This was the sole reason for its exclusion.

Great that you’ve added in some literature regarding men/boys/non-menstruators however the point that I wanted to get at was that it can be these groups that also act as a barrier to schoolgirls’ attendance through teasing or shaming.

**Authors ‘Response**

Dear reviewer the comment is well received. The following statement is included in line #389-392 on the current version which may address the concern about the effect of teasing by boys on school attendance and participation.

“Approximately 13% of Tanzanian girls have encountered period teasing, while over 80% expressed fear of being teased, particularly by male classmates. This results in reduced school attendance, participation, and concertation in class.”

I don’t think changing the line to ‘most religious beliefs’ avoids generalization either… this is a sensitive point or I understand what you’re getting at… but I think what you’re saying could be misinterpreted. Religion is interpreted and enacted differently across the world. For example in the UK, I don’t think many Christians would feel stigma towards menstruation because of what is written in the Bible. But I understand that other cultures might. What you can say instead which is factually true is that in certain religious texts menstruation is presented as making the menstruator impure or unclean. I would be particular about using the exact wording – so much of religious text is down to interpretation.

**Authors ‘Response**

This is well accepted and the statement “Menstrual restrictions and cultural taboos are often rooted in most religious beliefs and untrue cultural assumptions” Is replaced with following statement on the current version (line #395-397)

“In certain religious texts, menstruation is often framed as making the menstruator impure or unclean, leading to menstrual restrictions, shame, and taboos”

I feel that for this work to be relevant, contemporary, and self-aware, the authors should take a little more time to contemplate these last points. It would also be good to have a statement from the authors on why this paper is important and what new contribution to the discussions on MHM it brings.

**Authors ‘Response**

The contribution of the study to the discussion on MHM is highlighted as follows in the strength section (line #425-434)

The review also has several strengths, providing an extensive summary of English-language evidence. It offers valuable insights by presenting a comprehensive review of English-language trial studies that evaluate the effect of menstrual hygiene management interventions on various aspects of schoolgirls' lives. With a large sample size from multiple countries, the study covers a broad range of interventions, including puberty education, distribution of menstrual supplies, and integration of menstrual health topics into school curriculums. The findings not only underscore the positive effects of these interventions, such as increased school attendance and enhanced menstrual hygiene knowledge and attitudes, but also shed light on the challenges and limitations observed in certain studies. This review will also have great contribution in identifying research gaps for further studies.
