# Supplementary material for: Menstrual hygiene management interventions and their effects on schoolgirls’ menstrual hygiene experiences in low and middle countries: A systematic review
Source: PLoS One. 2024 Aug 22;19(8):e0302523. doi: 10.1371/journal.pone.0302523 (PMC11340951; doi:10.1371/journal.pone.0302523)
Supplement: Attachment — Submitted filename: Response to Reviewers.docx [file pone.0302523.s008.docx]

**Response to Reviewers**

1. If the authors have adequately addressed your comments raised in a previous round of review and you feel that this manuscript is now acceptable for publication, you may indicate that here to bypass the “Comments to the Author” section, enter your conflict-of-interest statement in the “Confidential to Editor” section, and submit your "Accept" recommendation.

Reviewer #1: All comments have been addressed

Reviewer #2: (No Response)

2. Is the manuscript technically sound, and do the data support the conclusions?

The manuscript must describe a technically sound piece of scientific research with data that supports the conclusions. Experiments must have been conducted rigorously, with appropriate controls, replication, and sample sizes. The conclusions must be drawn appropriately based on the data presented.

Reviewer #1: Yes

Reviewer #2: Yes

3. Has the statistical analysis been performed appropriately and rigorously?

Reviewer #1: N/A

Reviewer #2: Yes

4. Have the authors made all data underlying the findings in their manuscript fully available?

The [PLOS Data policy](http://www.plosone.org/static/policies.action#sharing) requires authors to make all data underlying the findings described in their manuscript fully available without restriction, with rare exception (please refer to the Data Availability Statement in the manuscript PDF file). The data should be provided as part of the manuscript or its supporting information, or deposited to a public repository. For example, in addition to summary statistics, the data points behind means, medians and variance measures should be available. If there are restrictions on publicly sharing data—e.g. participant privacy or use of data from a third party—those must be specified.

Reviewer #1: Yes

Reviewer #2: Yes

5. Is the manuscript presented in an intelligible fashion and written in standard English?

PLOS ONE does not copyedit accepted manuscripts, so the language in submitted articles must be clear, correct, and unambiguous. Any typographical or grammatical errors should be corrected at revision, so please note any specific errors here.

Reviewer #1: Yes

Reviewer #2: Yes

6. Review Comments to the Author

 **Reviewer #1**: I implore the authors to run their manuscript through a grammar-checking program (Grammarly, Paperpal, anything) or Chat GPT to improve the grammar and punctuation, especially in the first three pages. The authors have nicely addressed the substantive comments and have responded to specific grammatical errors pointed out by the reviewers. The manuscript still needs to be gone through line by line because parts of it are not well-edited. I do not consider it a good use of my professional time to provide a third round of input pointing out grammatical errors that could be addressed readily by the authors.

**Authors’ Response:**

Dear reviewer, this is valued concern. The necessary grammar and spelling errors are fixed throughout the document on the current version manuscript.

**Reviewer #2:** Thanks for addressing the comments. There are just a couple tinier things to address and then I’m happy for the work to be published.

**1**. ‘frequently written from the perspective of a cis woman’ – although I completely agree with this perhaps it requires a reference? Maybe you could explain why you know this to be true as I’m not sure you’d actually be able to find a reference. You could say ‘Since gender-diverse persons make up such a small percentage of the population, it can be assumed that the vast majority of academic literature on MHM is written from the perspective of cis women’. Maybe I’m being unnecessarily pedantic here.

**Authors’ Response:**

Dear reviewer this comment is valued and is addressed as follows in line # 415 and the statement is replaced with the recommended way of rephrasing.

‘Since gender-diverse persons make up such a small percentage of the population, it can be assumed that most of the academic literature on MHM is written from the perspective of cis women’.

**2**. I think you would do well to read around the purpose of positionality statements and this would help to inform how to write one. I wasn’t looking for you just to state that you are Ethiopian but how that background and identity shapes and influences the research. For example, something like: I am a woman (?) who comes from Ethiopia, which is one of the countries studied in this paper. This gives me first-hand experience of what it’s like to be a menstruating schoolgirl in an LMIC. However, I am an outsider to the other countries studied in this paper, which may leave room for bias or misunderstandings in the interpretation of results. Something like this.

**Authors’ Response:**

Dear reviewer the comment is well noted and the positionality statement in line #128-136, is rephrased as**;**

“I (the first author) am a woman, a feminist, and an advocate for girls' education. I am currently pursuing a PhD in public health. I am from Ethiopia, which is one of the countries studied in this paper. This gives me first-hand experience of what it’s like to be a menstruating schoolgirl in an LMIC. However, I am an outsider to the other countries studied in this paper, which may leave room for bias or misunderstandings in the interpretation of results. In this systematic review, the researcher's standpoint influences the research approach and findings. This study advocates for accessible menstrual hygiene resources and aims to address the stigma surrounding menstruation. The conclusions are based on this perspective, and readers are encouraged to take this into account when interpreting the findings.”

3. Perhaps I’m being too cautious, but I still believe this could be interpreted as offensive to some readers ‘In certain religious texts, menstruation is often framed as making the menstruator impure or unclean, leading to menstrual restrictions, shame, and taboos’. To reiterate, so much religious text is down to interpretation, and this is what can cause the issues of negative views towards menstruation, not necessarily the words in the text itself. I don’t know if you’re religious or not, but this would be an instance where your positionality affects how you interpret data. For example, I’m not religious and therefore don’t understand or have association to any religious texts. I gave a lecture on MHM making the exact same point as you that religious texts e.g. The Quran says that menstruation is impure. This was of course from a translation from Arabic into English where meaning can get lost anyway. A Muslim student put their hand up to explain that this was a very negative interpretation of the text and that it doesn’t mean impure in a dirty way as was interpreted. I think it would be inoffensive but still make the same point to say ‘Certain religious texts can be interpreted to associate menstruation with impurity and uncleanliness, which leads to menstrual restrictions, shame, and taboos in some cultures’ you need a reference for this as well.

**Authors’ Response:**

Dear reviewers I agree with comments. The statement in line **Line #392-94**: “In certain religious texts, menstruation is often framed as making the menstruator impure or unclean, leading to menstrual restrictions, shame, and taboos”

Is replaced with

‘Certain religious texts can be interpreted to associate menstruation with impurity and uncleanliness, which leads to menstrual restrictions, shame, and taboos in some cultures. And reference is cited.
